# Dual‐Responsive Hydrogels Integrate Bioelectrical Stimulation and Piezo‐Photocatalysis for Regenerative Treatment of Infected Wounds

**DOI:** 10.1002/advs.77132

**Published:** 2026-08-17

**Authors:** Baoying Dai, Wenxuan Qie, Xiaoye Li, Jingben Zheng, Ao He, Heng Dong, Jiangjiang Yan, Hao Zhang, Ziheng Pan, Hang Yin, Chuyi Zhou, Rui Kong, Hao Wang, Binghan Dai, Yannan Xie, Zhiqun Lin

**Affiliations:** ^1^ State Key Laboratory of Flexible Electronics (LoFE) and Institute of Advanced Materials (IAM) Jiangsu Provincial Industrial Technology Engineering Center for Flexible Mechatronics and Energy Systems Jiangsu Key Laboratory of Smart Biomaterials and Theranostic Technology Nanjing University of Posts and Telecommunications Nanjing China; ^2^ Department of Chemical and Biomolecular Engineering National University of Singapore Singapore Singapore; ^3^ Nanjing Stomatological Hospital Affiliated Hospital of Medical School Institute of Stomatology Nanjing University Nanjing China; ^4^ School of Foreign Studies China University of Geosciences Beijing China

**Keywords:** photocatalytic therapy, piezocatalytic therapy, piezo‐phototronic effect, reactive oxygen species, wound healing

## Abstract

Infected wounds represent a formidable clinical challenge, as persistent bacterial colonization and disrupted bio‐signaling collectively hinder effective tissue repair. Conventional passive dressings lack the capacity to actively modulate this complex microenvironment. Here, we report a wireless, dual‐responsive hydrogel (PVA/P(VDF‐TrFE)/BiOClBr@PDA, PPBP) based on porous poly(vinyl alcohol) (PVA), piezoelectric poly(vinylidene fluoride‐trifluoroethylene) (P(VDF‐TrFE)), and polydopamine‐modified bismuth oxybromide chloride (BiOClBr@PDA). The constructed hydrogel orchestrates piezoelectric stimulation and piezo‐photocatalysis to simultaneously eradicate infection and accelerate tissue regeneration. Under concurrent ultrasonic and optical stimulation, this platform efficiently transduces mechanical and light energy into therapeutic electrical cues and reactive oxygen species (ROS). This dual‐mode activation enables enhanced antibacterial efficacy against diverse bacterial strains, significantly outperforming single‐mode treatments while maintaining favorable biocompatibility. In an infected wound model, the constructed composite hydrogel PPBP effectively inhibits bacteria and attenuates inflammatory responses via controlled ROS generation, while piezoelectric stimulation supports re‐epithelialization and collagen deposition. Together, these effects promote accelerated and structurally improved wound healing. This work establishes a paradigm for utilizing multi‐source energy‐responsive hydrogels as an active strategy for regulating infected wound environments and supporting functional skin regeneration.

## Graphical abstract

A wireless, dual‐responsive hydrogel (PVA/(PVDF‐TrFE)/BiOClBr@PDA) is developed to promote efficient regeneration of infected wounds by integrating piezoelectric stimulation with piezo‐photocatalytic pathways. Under simultaneous light and ultrasonic stimulation, the hydrogel generates therapeutic electrical cues and reactive oxygen species, disrupting bacterial biofilms, attenuating inflammatory responses, promoting re‐epithelialization and collagen deposition, and ultimately leading to rapid and nearly complete wound closure.

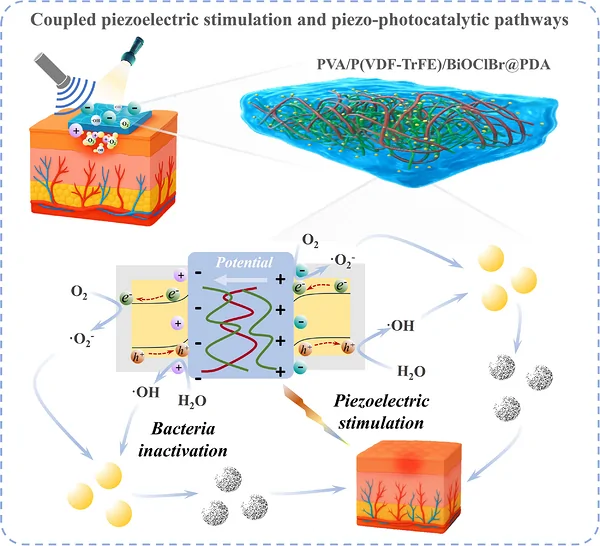

## 1 Introduction

The skin, as the body's largest organ and primary interface with the external environment, plays an indispensable role in maintaining physiological homeostasis. It provides a formidable shield against physical trauma, chemical hazards, and biological pathogens, while orchestrating critical functions such as thermoregulation, sensory perception, and metabolic detoxification [1, 2, 3]. When this defensive barrier is compromised by injury, underlying tissues become highly susceptible to microbial invasion and oxidative stress, often leading to delayed re‐epithelialization, non‐healing wounds, or systemic complications [4, 5]. Conventional therapeutic interventions, including surgical debridement, systemic antibiotics, and standard wound dressings, frequently fail to address these challenges, particularly in the context of multidrug‐resistant bacteria and the complex, dynamic wound microenvironment [6, 7, 8]. These limitations underscore an urgent need for advanced wound dressings capable of actively eradicating drug‐resistant biofilms, mitigating oxidative stress, and proactively stimulating tissue regeneration in a biocompatible manner. Photocatalytic therapy (PCT) has emerged as a promising noninvasive strategy [9, 10, 11, 12], in which semiconductor photocatalysts absorb light to generate electron‐hole pairs, which subsequently participate in redox reactions with ambient oxygen and water, yielding reactive oxygen species (ROS) such as hydroxyl radicals and superoxide anions that kill bacteria through oxidative damage without introducing resistance, while simultaneously shortening the inflammatory phase to facilitate tissue repair [13, 14, 15].

Despite its potential, the practical efficacy of PCT is often bottlenecked by the intrinsic rapid recombination of photogenerated electron‐hole pairs in conventional single‐component photocatalysts, limiting ROS production [16, 17]. The piezo‐phototronic effect, which synergistically couples piezoelectricity, semiconductor properties, and photoexcitation, offers a powerful strategy to overcome this limitation [18, 19]. Mechanical deformation, such as ultrasound or movement, induces a built‐in electric field in piezoelectric semiconductors, promoting carrier separation and amplifying catalytic efficiency [20, 21]. Beyond enhancing ROS generation, the piezoelectric field itself mimics endogenous bioelectricity, modulating fibroblast proliferation, collagen deposition, and tissue remodeling to accelerate wound closure. However, translating piezoelectric‐photocatalytic materials into clinical wound care has been hindered by rigid or semi‐rigid composite films that exhibit poor gas and exudate permeability, weak adhesion to irregular wound beds, and mechanical mismatch with soft tissues [22, 23]. These constraints restrict ROS generation and effective electrical stimulation, underscoring the need for a hydrogel platform that combines high‐efficiency piezo‐phototronic effect with tissue‐conformal, porous, and biocompatible properties.

Herein, we report a multifunctional hydrogel poly(vinyl alcohol)/poly(vinylidene fluoride‐trifluoroethylene)/polydopamine‐modified bismuth oxybromide chloride (PVA/P(VDF‐TrFE)/BiOClBr@PDA, PPBP) that integrates synergistic piezoelectric stimulation with piezo‐photocatalytic therapy (Figure 1). The composite hydrogel is engineered by embedding a visible‐light‐responsive BiOClBr photocatalyst, modified with polydopamine (PDA), into a porous, biocompatible matrix of PVA and piezoelectric P(VDF‐TrFE). This design ensures excellent tissue adaptability, permeability, and efficient energy transduction. Electrical and photoelectrochemical characterizations reveal enhanced charge separation and ROS generation under concurrent light and ultrasonic stimulation. The in vitro results demonstrate potent antibacterial efficacy against *Staphylococcus aureus* (*S. aureus*), methicillin‐resistant *Staphylococcus aureus* (MRSA), and *Escherichia coli* (*E. coli*). Furthermore, the in vivo results reveal accelerated wound healing, as evidenced by enhanced collagen deposition and re‐epithelialization, confirming the PPBP hydrogel as a smart, safe, and highly efficient platform for infected wound management and tissue regeneration.

**FIGURE 1:**
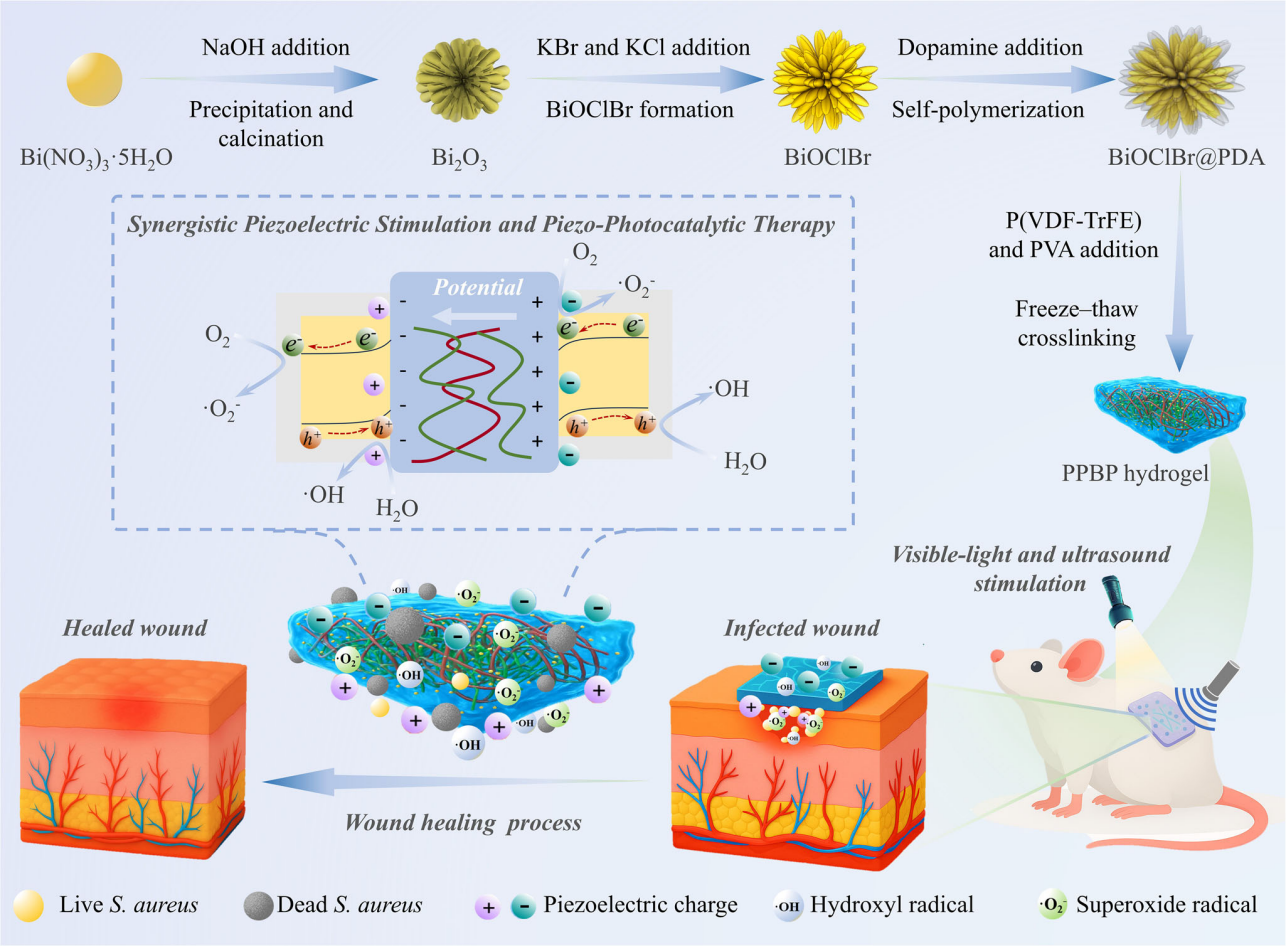
Schematic diagram of the fabrication process of PVA/P(VDF‐TrFE)/BiOClBr@PDA (PPBP) hydrogel and its application. PPBP hydrogel accelerating infected wound healing through synergistic piezoelectric stimulation and piezo‐photocatalytic therapy.

## 2 Results and Discussions

### 2.1 Preparation and Characterization of Structurally Stable PPBP Hydrogels

To elucidate the synergistic effects of piezoelectric stimulation and piezo‐photocatalytic therapy in wound healing, a multifunctional composite hydrogel PPBP was fabricated (Figure 1). Initially, flower‐like Bi_2_O_3_ was synthesized as a sacrificial template, exhibiting characteristic x‐ray diffraction (XRD) peaks at 25.9°, 27.0°, 33.3°, 35.1°, 37.6°, 46.5°, and 48.6°, which can be well indexed to tetragonal Bi_2_O_3_ (JCPDS No. 41–1449) (Figure 2a) [24]. Subsequently, the obtained Bi_2_O_3_ was transformed into BiOClBr through a halide ion exchange process, followed by surface coating with PDA to obtain BiOClBr@PDA. As shown in Figure 2a, both BiOClBr and BiOClBr@PDA exhibited nearly identical XRD patterns due to the limited PDA loading. The pronounced diffraction peaks can be assigned to the characteristic crystal planes of BiOCl (JCPDS No. 09–0393) and BiOBr (JCPDS No. 85–0861), confirming the successful formation of the BiOClBr solid solution [25, 26]. Compared with pristine BiOCl, the diffraction peaks of BiOClBr presented a noticeable shift toward lower diffraction angles, which is mainly attributed to the partial substitution of smaller Cl^−^ ions (1.81 Å) by larger Br^−^ ions (1.96 Å) within the BiOCl lattice. Such lattice expansion further verifies the effective incorporation of Br^−^ into the crystal framework. Moreover, the diffraction peak positions of BiOClBr are located between those of pure BiOCl and BiOBr, suggesting the formation of a homogeneous BiOClBr solid‐solution structure rather than a simple physical mixture, which is expected to facilitate electronic structure modulation and enhance photocatalytic activity. This conclusion was further supported by x‐ray photoelectron spectroscopy (XPS) analysis (Figure 2b–f). In the Bi 4f spectrum (Figure 2c), the peaks at 164.6 eV and 159.3 eV correspond to Bi 4f5/2 and Bi 4f7/2, respectively, corroborating the presence of Bi^3+^ species [27]. The O 1s spectrum (Figure 2d) was deconvoluted into two peaks at 531.4 eV and 529.9 eV, which are exclusively attributed to surface‐adsorbed H_2_O and the [Bi_2_O_2_]^2+^ species in the layered structure [28]. The Br 3d spectrum (Figure 2e) exhibited two peaks at 69.4 eV and 68.3 eV, corresponding to Br 3d3/2 and Br 3d5/2, respectively. Similarly, the Cl 2p spectrum (Figure 2f) showed peaks at 199.6 eV and 198.0 eV, assigned to Cl 2p1/2 and Cl 2p3/2 spin‐orbit components [29]. These results corroborate the successful formation of the BiOClBr solid solution and the stable incorporation of Br and Cl ions within the Bi‐O layered framework.

**FIGURE 2:**
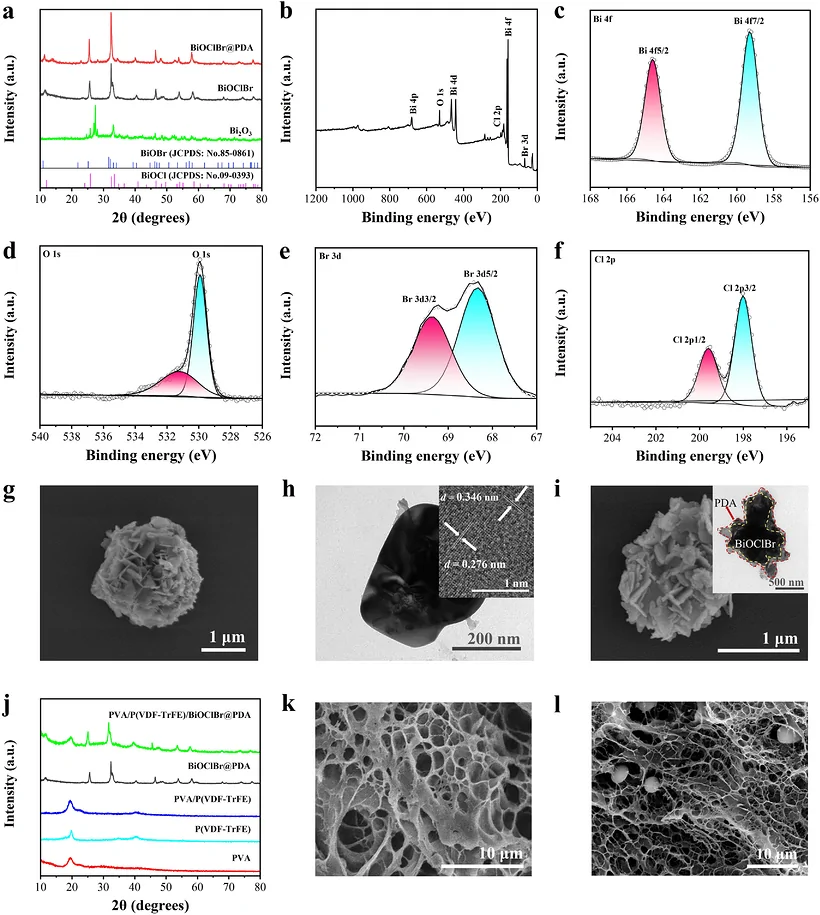
Structural and morphological characterization of the prepared samples. (a) XRD patterns of Bi_2_O_3_, BiOClBr, and BiOClBr@PDA powders. (b) XPS survey spectrum of BiOClBr and the corresponding high‐resolution spectra of (c) Bi 4f, (d) O 1s, (e) Br 3d, and (f) Cl 2p. (g) SEM image of BiOClBr nanoflower. (h) TEM and HRTEM (inset) images of a single BiOClBr nanosheet. (i) SEM and TEM (inset) images of BiOClBr@PDA. (j) XRD patterns of the prepared hydrogel samples. SEM images of (k) PVA/P(VDF‐TrFE) hydrogel and (l) PPBP composite hydrogel containing 0.1 g of BiOClBr@PDA.

The scanning electron microscopy (SEM) and transmission electron microscopy (TEM) images of BiOClBr revealed a uniform hierarchical nanoflower morphology composed of loosely assembled ultrathin nanosheets, forming a porous three‐dimensional architecture (Figure 2g,h). Such a structure is advantageous for enhancing light harvesting and exposing abundant reactive sites, thereby promoting photocatalytic activity. The high‐resolution TEM (HRTEM) image of BiOClBr displayed two distinct lattice fringes with interplanar spacings of 0.276 and 0.346 nm, which can be indexed to the (110) and (101) crystal planes of the tetragonal BiOClBr phase, respectively. The 0.276 nm spacing is consistent with the characteristic (110) planes of BiOCl (0.275 nm) and BiOBr (0.278 nm), while the 0.346 nm spacing corresponds well to the (101) plane [30]. These subtle lattice distortions further substantiate the formation of a mixed‐halide solid solution rather than a simple physical mixture. As shown in Figure 2i, the BiOClBr@PDA sample exhibited a distinct core‐shell structure, in which a thin PDA coating uniformly encapsulated the BiOClBr nanoflower. The presence of this conformal PDA layer was further verified by TEM observation, confirming the successful surface modification.

Based on the successful synthesis of BiOClBr@PDA, the composite was incorporated into biocompatible PVA and piezoelectric P(VDF‐TrFE) to construct a multifunctional hydrogel PPBP. As shown in Figure 2j, no distinct diffraction peaks were observed in the XRD pattern of the pure PVA hydrogel, indicating its amorphous nature. In contrast, a characteristic diffraction peak appearing at 19.9° was observed in both P(VDF‐TrFE) and PVA/P(VDF‐TrFE) samples, corresponding to the β‐phase of P(VDF‐TrFE), which is responsible for its intrinsic piezoelectric property. All characteristic peaks of both P(VDF‐TrFE) and BiOClBr@PDA were clearly retained in the XRD pattern of the PPBP hydrogel, indicating that the freeze–thaw fabrication process did not alter the crystal structures of either component. The well‐preserved β‐phase of P(VDF‐TrFE) suggests that the composite hydrogel maintains excellent piezoelectric performance after hybridization. Furthermore, this conclusion was corroborated by Fourier transform infrared (FT‐IR) analysis (Figure S1), where distinct absorption bands at approximately 841 and 1270 cm^−^^1^ were observed, corresponding to the vibrational mode characteristic of the β‐phase in P(VDF‐TrFE). It should be noted that in comparison with PVA/P(VDF‐TrFE), PPBP hydrogel presents more obvious absorption intensity at 841 and 1270 cm^−1^, which is attributed to BiOClBr@PDA serving as a nucleating agent to promote the formation of β‐phase within P(VDF‐TrFE) [31, 32, 33]. These results collectively corroborate the successful integration of BiOClBr@PDA within the piezoelectric polymer matrix to form a structurally stable multifunctional hydrogel.

As shown in Figure 2k–l, both the PVA/P(VDF‐TrFE) and PPBP hydrogels exhibited interconnected porous architectures, indicating that the incorporation of BiOClBr@PDA nanoparticles did not disrupt the intrinsic polymeric network of PVA and P(VDF‐TrFE). This three‐dimensional porous structure would endow the hydrogel with excellent breathability and water absorption capacity, facilitating oxygen and ion diffusion during the catalytic process and thereby promoting the generation of ROS. Compared with the pristine PVA/P(VDF‐TrFE) hydrogel, the SEM image of PPBP revealed the presence of spherical particles anchored on the pore walls, which can be attributed to the embedded BiOClBr@PDA nanoparticles (Figure 2l). These particles provide active sites and light‐responsive centers, enabling efficient photocatalytic performance. The improved interfacial compatibility is ascribed to the presence of the PDA coating on BiOClBr, which serves as an effective interfacial bridge. The low surface energy of PDA minimizes the interfacial tension between BiOClBr and P(VDF‐TrFE), while the hydrogen bonding between the fluorine atoms in the CF_2_ groups of P(VDF‐TrFE) and the hydrogen atoms in the amino groups of PDA strengthens interfacial adhesion. This synergistic interaction enhances nanoparticle dispersion and stability within the PVA/P(VDF‐TrFE) matrix, ensuring the uniform microstructure and multifunctional integrity of the PPBP hydrogel.

To comprehensively evaluate the mechanical and viscoelastic properties of the hydrogel, oscillatory rheological measurements and uniaxial tensile tests were systematically performed (Figures S2 and S3). Within the low‐strain regime, the storage modulus (G′) consistently exceeded the loss modulus (G″), indicating the predominance of elastic behavior and confirming the formation of a stable, well‐integrated three‐dimensional network. As the applied strain increased, G′ gradually declined and eventually intersected with G″ at approximately 10% strain, marking the critical strain point at which network integrity begins to deteriorate and the material transitions from a solid‐like to a liquid‐like state. This behavior reflects the progressive disruption of physical crosslinks under large deformation. Consistent with the rheological findings, tensile tests further demonstrated that the PPBP hydrogel possesses excellent mechanical robustness and stretchability, with an elongation at break of up to ∼400% and a maximum tensile stress of approximately 0.7 MPa. Notably, the stress–strain curves exhibited a smooth and continuous profile without abrupt fracture, suggesting a relatively homogeneous network architecture capable of effectively dissipating applied stress. Such energy dissipation is likely attributed to reversible interactions and dynamic crosslinking within the hydrogel matrix. Taken together, these results highlight that the hydrogel achieves an optimal balance between mechanical strength and deformability. It maintains structural integrity under small deformations while accommodating substantial strain without catastrophic failure. These combined viscoelastic and mechanical properties are particularly advantageous for wound dressing applications, as they enable intimate conformability to irregular tissue surfaces and ensure mechanical resilience under dynamic physiological conditions.

Furthermore, the structural stability of the PPBP hydrogel was further evaluated by monitoring its weight loss after immersion in phosphate buffer saline (PBS). As shown in Figure S4, the PPBP hydrogel exhibited a relatively slow degradation behavior, with only approximately 21.8% weight loss after 15 days of immersion. This moderate mass loss was mainly attributed to the dissolution of uncrosslinked PVA chains and the desorption of low‐molecular‐weight PVA components [34, 35]. To further elucidate the origin of the weight loss and assess the structural integrity of the hydrogel, XRD and FT‐IR analyses were performed on the PPBP hydrogel after immersion in PBS for 15 days. As shown in Figure S5a, no obvious changes were observed in the characteristic diffraction peaks of BiOClBr, P(VDF‐TrFE), or PVA, indicating that these components maintained excellent structural stability during PBS treatment. The preserved crystal structure of BiOClBr could be attributed to the protective PDA coating, which effectively inhibited structural deterioration in the aqueous environment, while the intrinsic stable chemical bonding within P(VDF‐TrFE) and PVA also contributed to the overall robustness of the hydrogel network [36, 37]. Consistently, the FT‐IR spectra before and after PBS immersion exhibited negligible variations in the characteristic functional group signals (Figure S5b), further confirming the preservation of the main chemical composition and structural integrity of the PPBP hydrogel. Furthermore, after partial dissolution of uncross‐linked and low‐molecular‐weight PVA components, the hydrogel became easier to detach from the skin and could be readily removed by gentle rinsing without the need for additional mechanical peeling force (Figure S6). Such a mild and non‐invasive removal process is beneficial for minimizing secondary damage to the newly formed epithelial tissue and reducing discomfort during dressing replacement. Collectively, these results demonstrate that the PPBP hydrogel possesses suitable structural stability, controllable degradation behavior, and favorable removability, highlighting its potential for practical wound‐healing applications.

### 2.2 Characterization of Piezo‐Photocatalytic Performance

The optical absorption behaviors of the prepared samples were investigated using UV–vis diffuse reflectance spectroscopy (Figure 3a). The pristine PVA hydrogel exhibited negligible absorption within the 250–850 nm range, indicating its intrinsic optical transparency. In contrast, the introduction of P(VDF‐TrFE) slightly enhanced the absorption intensity in the 250–550 nm region, which can be attributed to the increased light scattering induced by the polymeric blend. The BiOClBr@PDA powder showed a pronounced absorption edge at approximately 400 nm, consistent with previously reported data, confirming its visible‐light responsiveness [38]. The PPBP hydrogel displayed an absorption profile similar to that of BiOClBr@PDA, implying that the incorporation of BiOClBr@PDA endows the hydrogel with efficient light‐harvesting capability in the visible light region, which is beneficial for subsequent photo‐driven catalytic processes.

**FIGURE 3:**
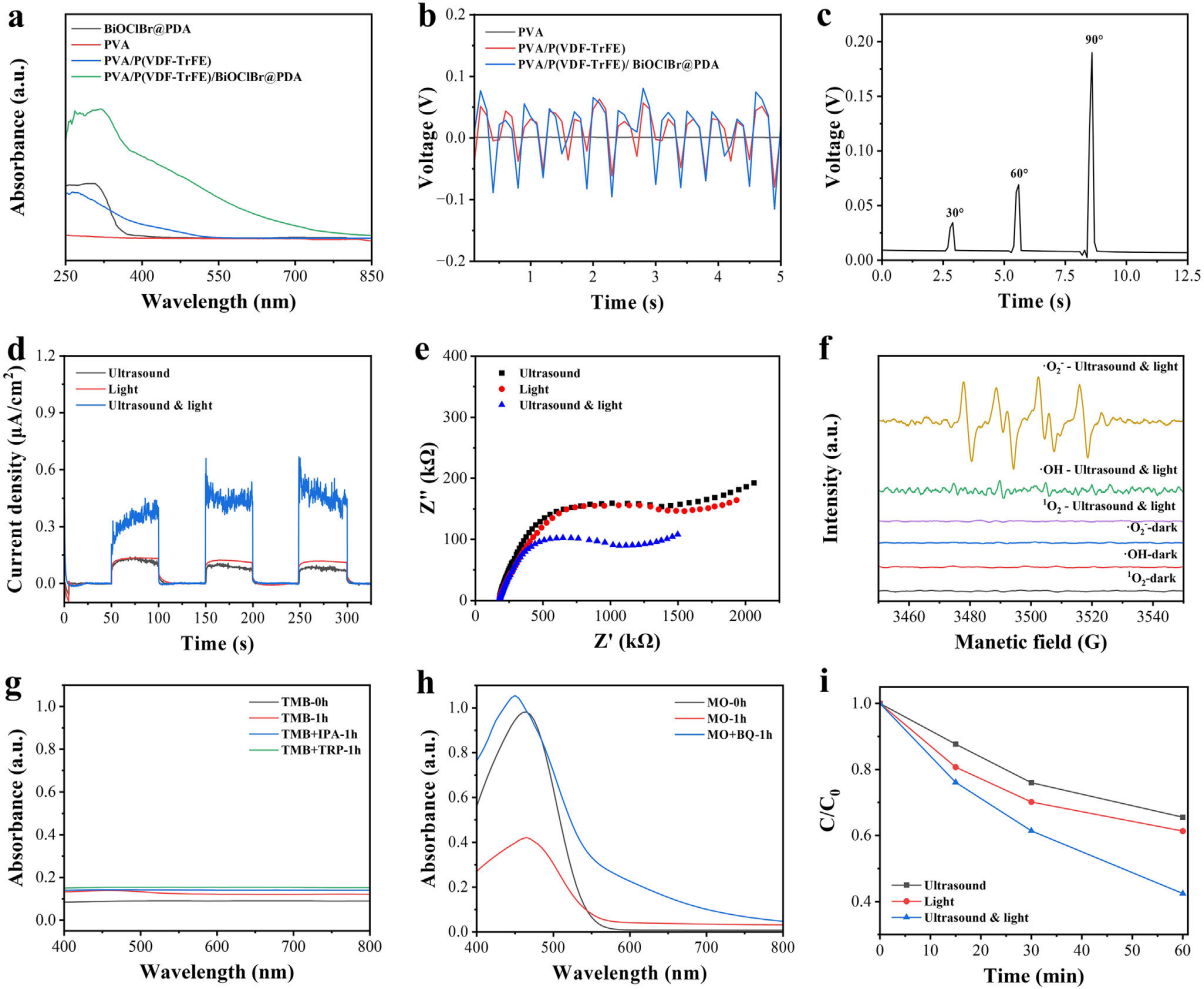
Optical absorption, piezoelectric property, charge carrier behaviors, and ROS generation performance of the prepared samples. (a) UV–vis absorption spectra. (b) Piezoelectric potential outputs of PVA, PVA/P(VDF‐TrFE), and PPBP hydrogels under ultrasonic stimulation. (c) Piezoelectric potential output of PPBP under finger bending at different angles. (d) Current density and (e) EIS Nyquist plots of PPBP hydrogel under various conditions. (f) ESR spectra of ·O^2−^, ·OH, and ^1^O_2_ detected by PPBP hydrogel. (g) Light absorbance spectra of TMB treated with PPBP under light and ultrasound for 1 h: original TMB (TMB‐0 h), TMB (TMB‐1 h), TMB with IPA (TMB‐IPA‐1 h), and TMB with TRP (TMB‐TRP‐1 h). (h) Light absorbance spectra of MO degradation under light and ultrasound: original MO (MO‐0 h), MO with PPBP for 1 h (MO‐1 h), and MO with PPBP in the presence of BQ for 1 h (MO+BQ‐1 h). (i) Degradation curves of MO solution catalyzed by PPBP hydrogel.

The piezoelectric properties of the hydrogels were further evaluated under ultrasonic excitation (Figure 3b). As expected, the PVA hydrogel exhibited no measurable potential output owing to its nonpiezoelectric nature. Differently, the PVA/P(VDF‐TrFE) and PPBP hydrogels generated distinct peak‐to‐peak potential outputs of approximately 120 mV and 160 mV, respectively, implying the successful introduction of piezoelectric functionality into the composite hydrogels. The significantly higher potential output of the PPBP hydrogel compared with PVA/P(VDF‐TrFE) can be ascribed to the synergistic role of BiOClBr@PDA. Specifically, BiOClBr@PDA acts as both a strain concentration center that amplifies the mechanical deformation under ultrasonic stimulation, and as a nucleating agent that promotes the formation of the piezoelectric β‐phase in P(VDF‐TrFE) (as demonstrated by the FT‐IR result) and thereby enhances the intrinsic piezoelectric response of the composite. To further assess its practical piezoelectric performance under dynamic mechanical deformation, the PPBP hydrogel was attached to a finger, and its output potential was monitored during bending at 30°, 60°, and 90°, respectively (Figure 3c). The output voltage increased progressively with the bending angle, demonstrating that larger mechanical strains lead to stronger piezoelectric signals. The piezoelectric potential outputs recorded under a defined mechanical force, generated by dropping a 200 g weight from a height of 5 cm, further demonstrate the superior piezoelectric performance of the PPBP hydrogel (Figure S7), enabling its effective utilization for self‐powered piezo‐photocatalysis driven by daily mechanical motions and ambient light.

To gain deeper insight into the underlying carrier separation and transfer dynamics, photoelectrochemical analyses, including transient current response and electrochemical impedance spectroscopy (EIS), were conducted under light irradiation, ultrasonic excitation, and their combination (Figure 3d,e). The PPBP hydrogel exhibited the highest current density (∼0.45 µA cm^−2^, piezo‐photocurrent) under dual stimulation, significantly exceeding that under individual light (∼0.12 µA cm^−2^, photocurrent) or ultrasound (∼0.11 µA cm^−2^, piezocurrent) excitation. Notably, the piezo‐photocurrent output under combined excitation surpassed the sum of the individual currents, demonstrating a strong synergistic effect between piezoelectric polarization and photoinduced carrier separation [39]. Correspondingly, the Nyquist plots revealed a smaller semicircular arc under dual stimulation, indicating reduced charge‐transfer resistance and more efficient carrier migration. These observations substantiate that the piezoelectric field generated by the polarized P(VDF‐TrFE) phase could effectively facilitate the spatial separation of photogenerated electron–hole pairs in BiOClBr@PDA, driving electrons and holes to migrate toward opposite directions and thereby suppressing their recombination under dual stimulation.

Electron spin resonance (ESR) spectroscopy was employed to identify the specific ROS generated by the PPBP hydrogel under mechanical strain and light irradiation (Figure 3f). In the absence of ultrasound and light, no ESR signals corresponding to ROS were detected. However, upon simultaneous ultrasound and light stimulation, distinct signals of superoxide radicals (·O_2_^−^) and weak hydroxyl radicals (·OH) were observed, while singlet oxygen (^1^O_2_) signals remained negligible. These findings demonstrate that the PPBP hydrogel effectively produces ROS under dual stimuli, with ·O_2_^−^ as the predominant species, which contributes to bacterial inactivation through oxidative damage. To further verify the ROS generation capability of PPBP hydrogel, radical trapping experiments were performed using 3,3′,5,5′‐tetramethylbenzidine (TMB) and methyl orange (MO) as indicator probes. TMB was used to detect ·OH and ^1^O_2_, forming a blue oxidized intermediate, while MO was employed to monitor ·O_2_^−^ through its degradation to colorless products. The negligible differences in the absorbance spectra of TMB solutions observed before and after treated with light and ultrasonic stimulation further suggest the minimal generation of ·OH and ^1^O_2_ (Figure 3g). Figure 3h and Figure S8 showed a pronounced decrease in absorbance after 1 h of either solo light, solo ultrasonic, or dual stimulation, which was significantly suppressed upon the addition of ·O_2_^−^ scavenger benzoquinone (BQ). These results collectively verify that ·O_2_^−^ as the dominant ROS can be produced by the PPBP hydrogel via photocatalysis, piezocatalysis, and piezo‐photocatalysis processes, respectively. Furthermore, the markedly higher MO degradation efficiency observed under combined ultrasonic and light stimulation compared to that under individual conditions highlights a pronounced synergistic effect between piezocatalysis and photocatalysis in ROS generation (Figure 3i).

Moreover, to optimize the BiOClBr@PDA content within the PPBP hydrogel for achieving superior piezo‐photocatalytic performance, a series of PPBP hydrogels containing different amounts of BiOClBr@PDA were synthesized (Figures S9 and S10). All samples exhibited nearly identical XRD patterns and comparable morphological features to those of the PPBP hydrogel with 0.1 g of BiOClBr@PDA, indicating that the incorporation of BiOClBr@PDA powders into the PVA/P(VDF‐TrFE) matrix does not alter the crystalline phase or porous network structure of the hydrogel. The catalytic degradation of MO over PPBP hydrogels with varying BiOClBr@PDA contents was evaluated under simultaneous light and ultrasonic stimulation (Figure S11). The degradation efficiency initially increased with the BiOClBr@PDA loading and reached its maximum at 0.1 g. However, a further increase to 0.15 g resulted in a gradual decline in catalytic performance. This trend can be attributed to two competing factors: on the one hand, an appropriate BiOClBr@PDA content enhances both piezoelectric and photocatalytic activities by serving as strain concentration centers and active catalytic sites [40]; on the other hand, excessive BiOClBr@PDA tends to agglomerate, disrupting the uniform hydrogel network and impeding light penetration, thereby reducing the effective utilization of catalytic sites [41]. These results highlight the importance of compositional optimization in achieving high‐efficiency piezo‐photocatalysis. Consequently, the PPBP hydrogel containing 0.1 g of BiOClBr@PDA was selected as the optimal formulation for subsequent antibacterial and wound healing investigations.

### 2.3 In Vitro Antibacterial Activity and Biocompatibility Evaluation

Effective eradication of bacterial colonization is a prerequisite for shifting infected wounds from the inflammatory phase to the proliferative phase. To dissect the individual and combined contributions of optical and mechanical stimulation, five groups were established: PBS (Group 1), PPBP hydrogel without stimulation (Group 2), PPBP hydrogel under light irradiation (Group 3), PPBP hydrogel under ultrasonic stimulation (Group 4), and PPBP hydrogel under combined light and ultrasonic irradiation (Group 5). Qualitative colony‐forming assays of *S. aureus* (Figure 4a) showed that the pristine hydrogel (Group 2) exhibited a bacterial density comparable to the PBS control, indicating negligible intrinsic bactericidal activity of the material itself. In contrast, Groups 3 and 4 displayed visibly reduced colony counts. The decreased bacterial survival in Group 3 is primarily attributed to photocatalytically generated ROS, whereas that in Group 4 is mainly ascribed to ROS produced via piezocatalysis, together with antibacterial effects induced by localized piezoelectric stimulation. Notably, Group 5 achieved nearly complete eradication of bacterial colonies. To quantitatively evaluate this effect, bacterial inactivation efficiencies were further calculated (Figure 4b). The pristine hydrogel exhibited only a marginal inactivation efficiency of ∼13.6%. Under single‐stimulation conditions, the inactivation efficiencies increased to 51.8% under light irradiation and 37.2% under ultrasound exposure, respectively. Remarkably, the combined light and ultrasound treatment yielded a markedly enhanced inactivation efficiency of 97.7%, indicating a non‐additive enhancement over single‐mode treatments. This nonadditive enhancement clearly indicates a strong synergistic effect rather than a simple superposition of the two processes, which can be attributed to the synergistic effect between piezoelectric polarization and piezo‐photocatalytic reactions. Specifically, the mechanical deformation‐induced internal electric field within the P(VDF‐TrFE) matrix facilitates charge separation in BiOClBr, thereby boosting ROS generation, while the simultaneous piezoelectric stimulation further contributes to bacterial membrane disruption, collectively leading to substantially improved antibacterial performance. Further mechanistic insight was obtained by confocal laser scanning microscope (CLSM) and SEM characterizations. Live/dead staining (Figure 4c) revealed that the dual‐stimulation group was dominated by red fluorescence with minimal green signal, confirming extensive bacterial death. SEM imaging (Figure 4d) corroborated these observations: bacteria exposed to single‐mode stimulation displayed mild membrane wrinkling, whereas those subjected to dual stimulation exhibited severe structural collapse and evident membrane perforation. This irreversible damage is likely driven by the combined assault of ROS bursts and piezoelectrically induced microfields, which together compromise membrane integrity and promote leakage of intracellular components.

**FIGURE 4:**
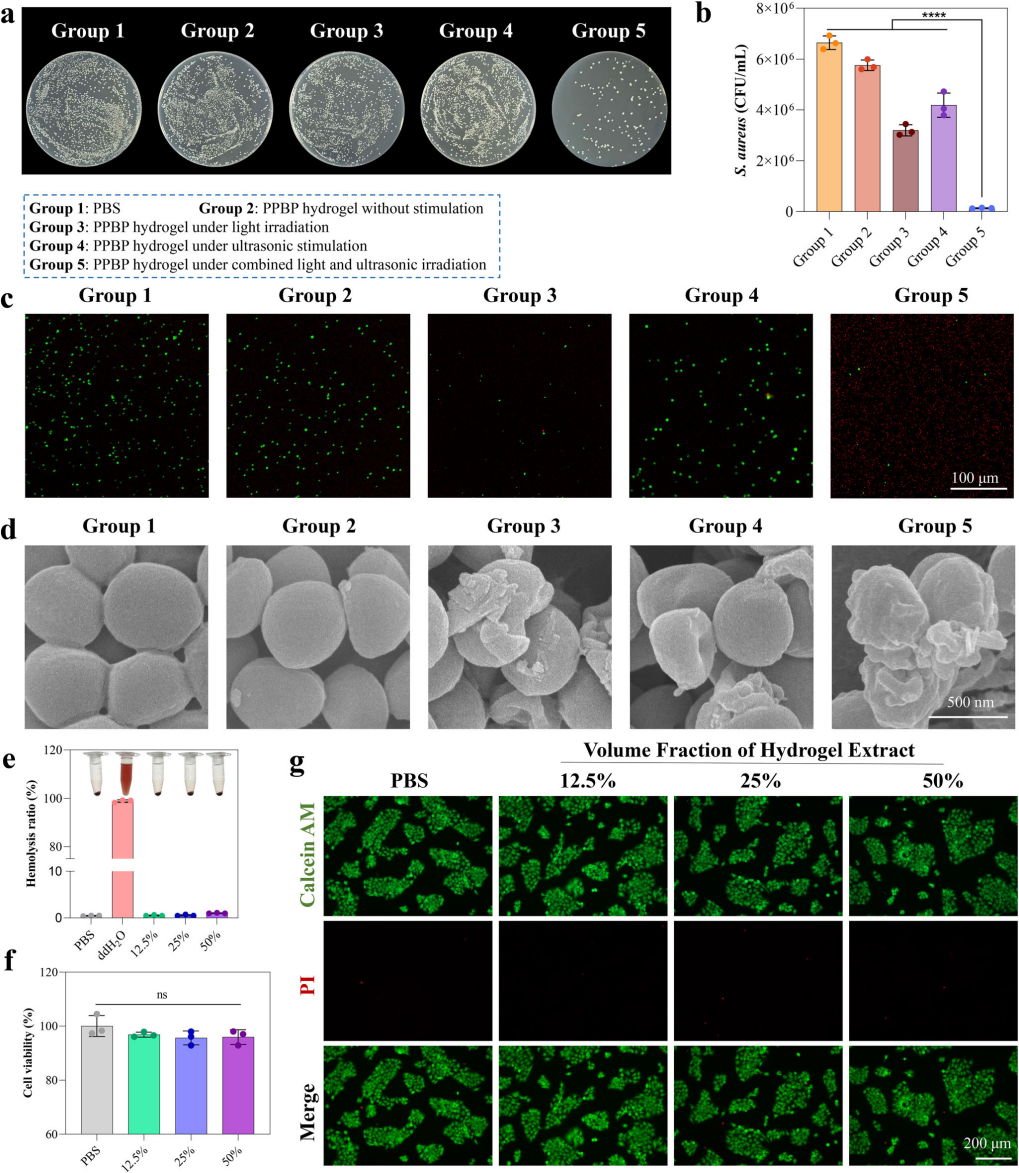
In vitro antibacterial efficacy and biocompatibility evaluation of PPBP hydrogel. (a) Representative photographs of *S. aureus* colonies on agar plates after treatment with PBS (Group 1), PPBP hydrogel (Group 2), PPBP and light irradiation (Group 3), PPBP and ultrasonic stimulation (Group 4), and PPBP with combined light and ultrasonic stimulations (Group 5). (b) Quantitative analysis of *S. aureus* viability (CFU/mL) corresponding to treatments in (a). Data are presented as mean ± S.D. (*n* = 3, ^****^ *p* < 0.0001). (c) CLSM images of *S. aureus* stained with a live/dead kit after various treatments. Live bacteria appear green and dead bacteria appear red. (d) SEM images revealing morphological alterations of *S. aureus* cells under the indicated treatments. (e) Hemolysis assay results. (f) Viability of HaCaT cells after incubation with different concentrations of PPBP hydrogel extracts, assessed by CCK‐8 assay. (g) Representative live/dead fluorescence images of HaCaT cells co‐cultured with PPBP extracts. Live cells are stained green (Calcein AM), and dead cells are stained red (PI).

To elucidate the potential contribution of the photothermal effect of PDA to the antibacterial performance, the photothermal behavior of the PPBP hydrogel was further investigated (Figure S12). Under periodic light irradiation (4 min on and 1 min off), the temperature of the hydrogel increased gradually and stabilized within a narrow range of approximately 37–38.6°C. This moderate thermal response can be partly attributed to the limited amount of PDA within the hydrogel and the high content of water in the hydrogel, which facilitates efficient heat dissipation and suppresses significant temperature accumulation. It should be noted that such a limited temperature rise is well below the threshold (∼50°C) generally required for effective photothermal bactericidal activity, indicating that direct photothermal sterilization is unlikely to be the dominant mechanism [42]. Nevertheless, although insufficient for direct bacterial inactivation, the mild temperature elevation may still contribute to the antibacterial process by enhancing ROS‐mediated activity, increasing bacterial susceptibility to oxidative stress, and improving the local microenvironment [43]. These auxiliary effects, in combination with piezo‐photocatalytic ROS generation and localized piezoelectric stimulation, synergistically contribute to the overall antibacterial performance of the PPBP hydrogel.

To further evaluate the antibacterial spectrum and potential clinical applicability of the hydrogel dressing, additional antibacterial assays were performed against methicillin‐resistant *Staphylococcus aureus* (MRSA) and *Escherichia coli* (*E. coli*), representing a clinically important multidrug‐resistant Gram‐positive strain and a typical Gram‐negative pathogenic bacterium, respectively. As shown in Figure S13, both MRSA and *E. coli* exhibited obvious reductions in viable bacterial colonies after hydrogel treatment under external stimulation. Quantitative colony‐forming unit analysis further confirmed that the antibacterial efficiencies against MRSA and *E. coli* followed a trend similar to that observed for *S. aureus*. In particular, the hydrogel combined with light and ultrasonic stimulation produced the most pronounced antibacterial effect, indicating that the hydrogel is not limited to Gram‐positive bacteria but also shows effective antibacterial activity against Gram‐negative bacteria and drug‐resistant strains.

In addition to planktonic bacteria, bacterial biofilms represent a major obstacle in the treatment of chronic and infected wounds. Mature biofilms are protected by extracellular polymeric substances, which can hinder antibacterial agent penetration and reduce bacterial susceptibility to treatment. To directly evaluate the biofilm clearance capacity of the hydrogel, an in vitro mature bacterial biofilm model was established, and crystal violet staining was used for both qualitative observation and quantitative biomass analysis. To avoid artificial biofilm disruption caused by direct contact between the hydrogel and the biofilm surface, a non‐contact Transwell‐like experimental setup was designed (Figure S14a). The crystal violet staining results demonstrated that the untreated biofilm maintained a dense and continuous structure, indicating successful formation of a mature bacterial biofilm. Among all groups, the hydrogel combined with light and ultrasonic stimulation exhibited the strongest biofilm clearance effect, confirming the synergistic contribution of photocatalytic and piezocatalytic activation. Interestingly, the ultrasound‐treated group showed a stronger biofilm removal effect than the light‐treated group in the mature biofilm model, which differed from the antibacterial trend observed in planktonic bacteria, where light stimulation produced a more pronounced bactericidal effect (Figure S14b). This phenomenon may be explained by the distinct structural characteristics of bacterial biofilms. Compared with planktonic bacteria, mature biofilms possess a compact extracellular polymeric matrix that limits the diffusion of antibacterial factors. Ultrasound can generate mechanical vibration, acoustic cavitation, and microstreaming effects, which may loosen the dense biofilm structure, promote detachment of the extracellular matrix, and enhance the penetration of ROS into deeper biofilm layers. Therefore, ultrasound stimulation may contribute more significantly to biofilm dispersion and biomass reduction, whereas light stimulation mainly contributes to direct ROS‐mediated bacterial killing.

Before moving toward in vivo application, it is essential to establish the biosafety of the PPBP hydrogel. Hemocompatibility was first assessed to evaluate its interaction with blood components. As shown in Figure 4e, the positive control (ddH_2_O) caused complete lysis of red blood cells (RBCs), yielding a uniformly red solution. In contrast, supernatants from PPBP hydrogel extrat (12.5%–50% v/v) remained clear and colorless, similar to the PBS negative control, indicating preservation of RBC integrity. Quantitative spectrophotometric analysis confirmed that the hemolysis ratios of all hydrogel extrat groups were below 5%, satisfying the standard criterion for acceptable hemocompatibility. Cytocompatibility was further evaluated using human immortalized keratinocytes (HaCaT) to mimic the cutaneous cellular microenvironment. CCK‐8 assays (Figure 4f) showed that HaCaT viability remained above 95% after incubation with the PPBP hydrogel extract. Live/dead staining (Figure 4g) demonstrated high cell viability and intact morphology across all hydrogel extract concentrations, as evidenced by predominant green fluorescence (live cells) and negligible red fluorescence (dead cells). Collectively, these results confirm that the PPBP hydrogel possesses excellent hemocompatibility and cytocompatibility. Such favorable biocompatibility is attributed to the biomimetic PVA‐based matrix and the stable encapsulation of catalytic components, establishing a robust safety profile and supporting its potential clinical translation as a wound management platform.

### 2.4 In Vivo Evaluation of Infected Wound Healing

Given the in vitro excellent antibacterial activity and favorable biocompatibility of the PPBP hydrogel, its in vivo wound‐healing performance was further assessed using a *S. aureus*–infected full‐thickness wound model. Wounds were treated under five conditions consistent with the in vitro antibacterial assays: PBS (Group 1), PPBP hydrogel without stimulation (Group 2), PPBP hydrogel with light irradiation (Group 3), PPBP hydrogel with ultrasonic stimulation (Group 4), and PPBP hydrogel with combined light and ultrasonic stimulations (Group 5). Throughout the healing period, wound area, bacterial burden, and histopathological changes were systematically monitored and recorded (Figure 5a). Representative photographs and corresponding quantitative analyses (Figure 5b,c) showed that, compared with Groups 1 and 2, all hydrogel groups with external stimulation exhibited accelerated wound closure. Notably, wounds in Group 5 (dual stimulation) were almost completely healed by the end of the treatment period, highlighting the strong wound‐regenerative capacity of the dual‐stimulated PPBP hydrogel. As shown in Figure 5c, the residual average wound areas in Groups 1, 2, 3, and 4 were 0.61, 0.62, 0.32, and 0.25 cm^2^, respectively, reflecting the contributions of photocatalytic therapy (Group 3) and piezocatalytic/electrical stimulation (Group 4). Notably, the average wound area in Group 5 was only 0.07 cm^2^, which is approximately 8.7‐, 8.8‐, 4.6‐, and 3.6‐fold smaller than those in Groups 1, 2, 3, and 4, respectively. These results clearly demonstrate that the combined piezoelectric stimulation and piezo‐photocatalytic therapy strategy markedly outperforms either single modality in promoting infected wound healing. To further evaluate antibacterial efficacy in vivo, wound tissues were collected and analyzed for bacterial burden, and colony‐forming units were quantified (Figure 5d,e). Compared with Groups 1 and 2, Groups 3, 4, and 5 exhibited substantial reductions in bacterial colonies, confirming the antibacterial effect of PPBP under external stimulation. Importantly, the bacterial concentration in Group 5 was approximately 1.58 and 1.61 orders of magnitude lower than those in Groups 3 and 4, respectively, corresponding to a sterilization efficiency of ∼98.4%. This pronounced antibacterial effect under dual stimulation underscores the potent piezoelectric stimulation and piezo‐photocatalytic sterilization capability of the PPBP hydrogel, which effectively suppresses bacterial proliferation, shortens the inflammatory phase, and thereby facilitates tissue regeneration and remodeling. Since ROS generation is considered the key mediator of the antibacterial activity of this system, direct in vivo evidence of local ROS production at the wound site was further required. To verify in vivo ROS generation, wound tissues were collected 12 h after treatment and stained with a ROS‐sensitive fluorescent probe. As shown in Figure S15, weak ROS signals were observed in the Groups 1 and 2, while stronger fluorescence appeared in the stimulation‐treated groups. Notably, the combined light and ultrasound treatment group showed the highest ROS fluorescence intensity, confirming that the PPBP hydrogel could effectively generate ROS locally at the wound site under dual stimulation.

**FIGURE 5:**
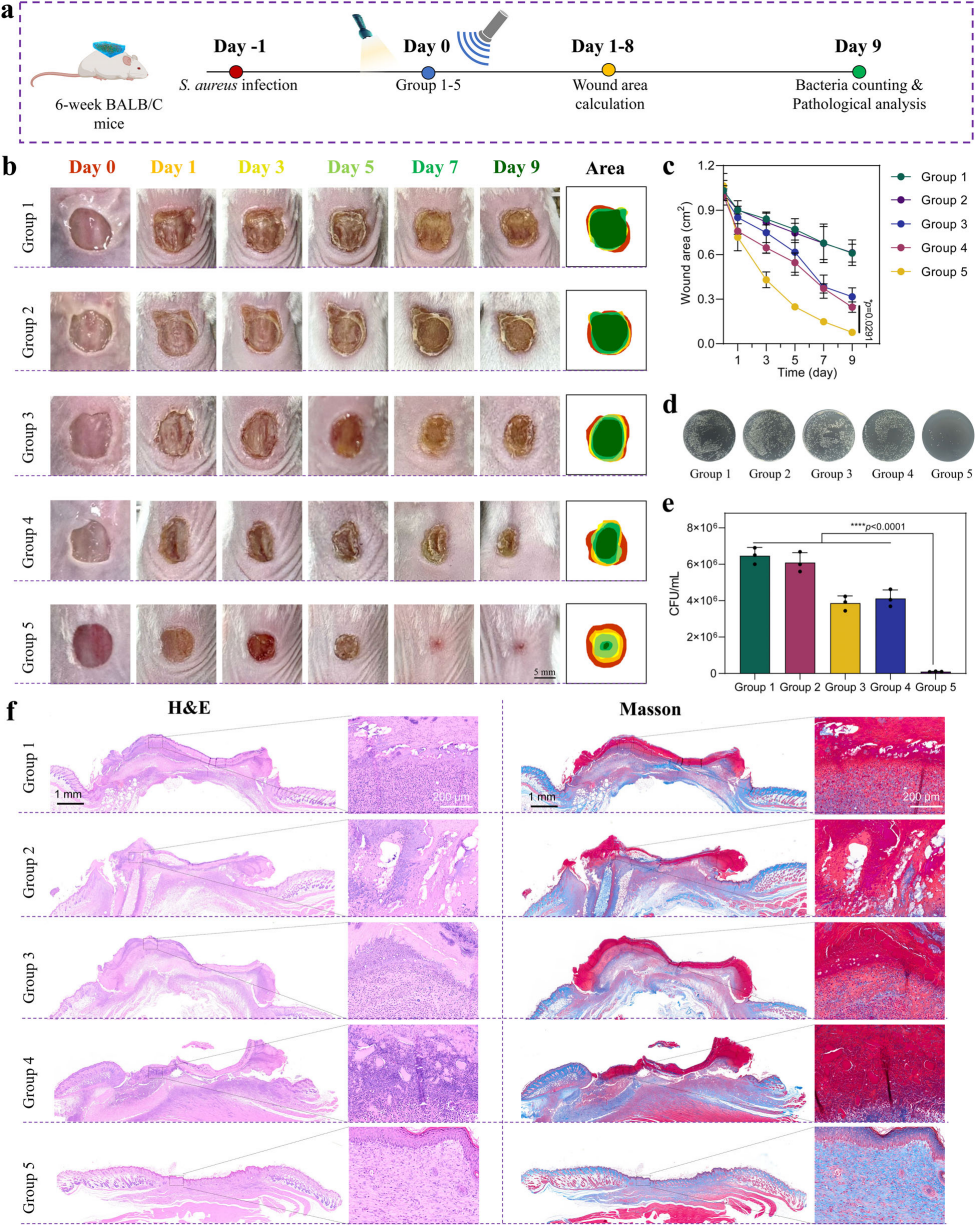
In vivo evaluation of PPBP hydrogel‐mediated infected wound healing. (a) Schematic of *S. aureus*‐infected mouse wound model and experimental treatment timeline from day 1 to day 9. (b) Representative photographs and corresponding wound tracings for five treatment groups: PBS (Group 1), PPBP hydrogel (Group 2), PPBP and light irradiation (Group 3), PPBP and ultrasonic stimulation (Group 4), PPBP with combined light and ultrasonic stimulations (Group 5) on days 0, 1, 3, 5, 7, and 9 post‐treatment. (c) Quantitative analysis of wound closure area (cm^2^) over time for each group (*n* = 6). (d) Representative photographs of *S. aureus* colonies on agar plates, cultured from wound tissues collected on day 9. (e) Quantitative analysis of viable *S. aureus* bacteria (CFU/mL) in wound tissues on day 9. Data are presented as means ± SD (*n* = 3, ^****^ *p* < 0.0001). (f) Representative optical micrographs of wound tissue sections harvested on day 9, stained with H&E (left panel) and Masson's trichrome (right panel) to visualize histological features and collagen deposition.

Histological analyses were performed to further evaluate the effects of different treatments on infected wound repair (Figure 5f). Hematoxylin and eosin (H&E) staining on Day 9 showed extensive inflammatory cell infiltration and incomplete, disorganized epithelial structures in the PBS and hydrogel‐only groups (Groups 1 and 2), indicating delayed wound repair. In contrast, Groups 3 and 4 exhibited reduced inflammatory infiltration and partial re‐epithelialization, suggesting that either light‐ or ultrasound‐induced catalytic activation could promote tissue repair to some extent. Notably, the wounds treated with PPBP hydrogel under combined light and ultrasonic irradiation (Group 5) displayed well‐developed granulation tissue, a continuous and uniform epithelial layer, and newly formed hair follicles, indicating more advanced wound healing and partial restoration of skin appendages. Masson's trichrome staining further confirmed improved extracellular matrix remodeling in Group 5, as evidenced by abundant, densely deposited, and well‐organized collagen fibers, whereas the other groups showed relatively sparse and disordered collagen deposition. Quantitative analysis also demonstrated that Group 5 exhibited the highest collagen deposition level, supporting its superior ability to promote extracellular matrix reconstruction (Figure S16).

To further investigate the regenerative mechanism, qRT‐qPCR analysis was performed to assess the expression of tissue repair‐related genes in wound tissues (Figure S17). Compared with the control group, Group 5 showed significantly upregulated mRNA levels of *Tgfb1*, *Arg‐1*, *Vegfa*, and *Col1a1*. The increased expression of *Tgfb1* and *Col1a1* suggests enhanced collagen synthesis and matrix remodeling, while the elevated *Vegfa* level indicates promoted angiogenesis. In addition, the upregulation of *Arg‐1* suggests the formation of a repair‐associated anti‐inflammatory microenvironment, which may facilitate the transition from the inflammatory phase to the proliferative phase of wound healing. Since angiogenesis is essential for nutrient supply and tissue reconstruction, we further evaluated the effect of different ultrasonic stimulation intensities on vascular regeneration in the PPBP hydrogel under combined light and ultrasonic irradiation. CD31 immunofluorescence staining showed that CD31 expression increased from 0 to 1 W/cm^2^, with the 1 W/cm^2^ group exhibiting the strongest fluorescence intensity (Figure S18). Further increasing the ultrasonic intensity to 2 W/cm^2^ did not lead to an additional increase in CD31 expression, indicating that 1 W/cm^2^ was sufficient to achieve an effective pro‐angiogenic response in this system.

To assess the systemic biosafety of the PPBP hydrogel, major organs, including the heart, liver, spleen, lung, and kidney, were harvested from noninfected mice treated with PBS or PPBP hydrogel under dual stimulation for histological examination. As shown in Figure S19, H&E staining revealed no obvious histopathological abnormalities, inflammatory infiltration, or tissue necrosis in any examined organs. The well‐preserved tissue architecture and cellular morphology indicate that the PPBP hydrogel, even under repeated light and ultrasound exposure, did not induce detectable systemic organ damage.

The long‐term biosafety and in vivo metabolic behavior of the PPBP hydrogel were further evaluated by routine blood analysis and Bi biodistribution assays. As shown in Figure S20, routine blood parameters, including white blood cells (WBC), red blood cells (RBC), monocytes (MON), mean corpuscular volume (MCV), platelets (PLT), and lymphocytes (LYM), remained within the normal physiological reference ranges in all treatment groups, indicating the absence of obvious hematological toxicity, systemic inflammatory response, or immune cell abnormalities. In addition, Bi distribution in major organs was quantified on Day 14 and Day 28 after treatment. As shown in Figure S21, Bi was mainly detected in the kidney, whereas only low levels were observed in the heart, liver, spleen, and lung. Importantly, the overall Bi concentration remained low at both time points, and no progressive accumulation was observed from Day 14 to Day 28. These results suggest limited systemic distribution and low long‐term retention risk of the Bi‐based component. The relatively higher Bi level in the kidney further indicates that renal clearance may participate in the elimination of Bi‐related components.

Finally, the local tissue safety of different ultrasonic stimulation parameters was evaluated using normal skin tissues. As shown in Figure S22, H&E staining demonstrated intact epidermal and dermal structures in the control group. Similarly, no obvious epidermal disruption, dermal necrosis, inflammatory cell infiltration, or subcutaneous tissue injury was observed after ultrasonic stimulation at 0.5, 1, or 2 W/cm^2^. The comparable tissue morphology among all groups indicates that ultrasonic stimulation within the tested range did not cause evident histological damage to normal skin tissues. Notably, the optimized intensity of 1 W/cm^2^ showed good tissue compatibility while effectively promoting wound angiogenesis, supporting the safety and rationality of this stimulation parameter for in vivo treatment.

Taken together, these results demonstrate that the dual‐stimulated PPBP hydrogel exerts its synergistic piezoelectric stimulation and piezo‐photocatalytic therapeutic effects locally at the wound site, efficiently eradicating bacteria, attenuating inflammation, enhancing epithelial regeneration, promoting collagen deposition, and ultimately accelerating wound closure, while maintaining excellent in vivo safety. This establishes PPBP as a promising multifunctional hydrogel platform with strong potential for clinical translation in the management of infected wounds.

### 2.5 Synergistic Mechanism of Piezoelectric Field and Piezo‐Photocatalytic Therapy

Building upon the aforementioned results, a mechanistic illustration of the synergistic piezoelectric stimulation and piezo‐photocatalytic therapy for enhanced wound healing is proposed and depicted in Figure 6. When the PPBP composite hydrogel is subjected to light irradiation, electrons (e^−^) in the valence band (VB) of BiOClBr are excited to its conduction band (CB), leaving behind positively charged holes (h^+^) in the VB. These photoexcited carriers subsequently migrate to the BiOClBr surface, where electrons are further transferred to the conductive PDA layer, facilitating spatial charge separation. The separated electrons and holes participate in photocatalytic redox reactions with surrounding oxygen and water molecules, generating ROS, such as ·O_2_^−^ and limited ·OH. However, the high carrier recombination rate severely restricts the ROS generation efficiency, leading to poor antibacterial performance (Figure 6a). Under ultrasonic stimulation, the P(VDF‐TrFE) phase within the hydrogel undergoes mechanical deformation, inducing a piezoelectric polarization field. As Figure 6b illustrated, this field redistributes internal charges, attracting negative and positive charges to regions of opposite polarization, thereby enabling piezocatalytic redox reactions between accumulated charges and surrounding molecules to generate ·O_2_^−^ and limited ·OH radicals. Nevertheless, because of the limited piezoelectric potential output, the piezocatalytic ROS generation and antibacterial efficiency remain modest when the piezoelectric effect acts alone.

**FIGURE 6:**
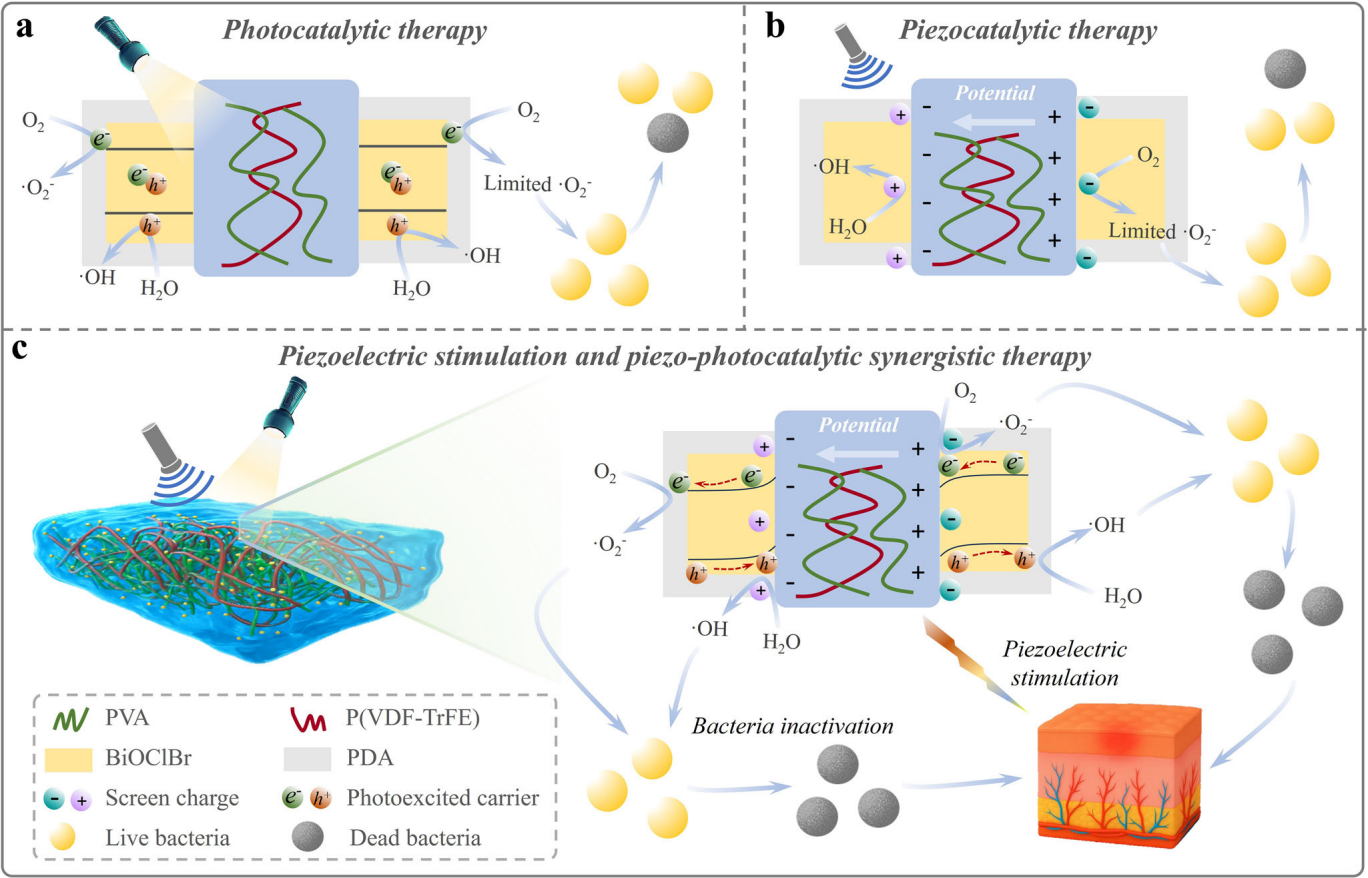
Schematic illustrating the mechanism by which the PPBP hydrogel synergistically promotes wound healing through piezoelectric field and piezo‐photocatalysis. Carrier separation and ROS generation under (a) light irradiation alone, (b) ultrasonic stimulation alone, and (c) combined light and ultrasonic irradiation.

Under combined light and ultrasonic stimulation, the strain‐induced piezoelectric polarization field generated by P(VDF‐TrFE) plays multiple synergistic roles in enhancing the therapeutic performance of the PPBP hydrogel. On the one hand, the piezoelectric polarization directly contributes to piezocatalytic ROS generation, thereby exerting antibacterial activity. On the other hand, the piezoelectric field serves as an internal driving force that promotes the directional separation and migration of photoexcited electron–hole pairs in BiOClBr@PDA, markedly suppressing charge recombination and consequently enhancing ROS production efficiency (Figure 6c). More importantly, beyond facilitating carrier migration, the piezoelectric potential can further regulate the interfacial electronic structure through the piezo‐phototronic effect. Specifically, the strain‐induced piezoelectric field generates interfacial band bending at the BiOClBr@PDA/P(VDF‐TrFE) heterointerface, which lowers the energy barrier for charge transfer and accelerates the separation and migration of photogenerated carriers. As a result, a larger proportion of charge carriers participates in interfacial redox reactions, thereby leading to significantly enhanced ROS generation and improved antibacterial efficiency under dual light‐ultrasound stimulation. In addition to its catalytic contribution, the piezoelectric field also provides beneficial electrical stimulation to the wound microenvironment, such as promoting collagen deposition, angiogenesis, and tissue regeneration [44]. Therefore, the synergistic integration of piezocatalysis, piezoelectric stimulation, and piezoelectric‐field‐enhanced photocatalysis enables a robust ROS burst for efficient bacterial eradication while simultaneously alleviating inflammation, facilitating epithelial regeneration, and promoting collagen deposition. Collectively, this synergistic mechanism between piezoelectric stimulation and piezo‐photocatalysis markedly amplifies the therapeutic efficacy of the PPBP hydrogel, ultimately accelerating the wound‐healing process.

## 3 Conclusions

In summary, we developed a multifunctional PPBP hydrogel that combines piezoelectric stimulation and piezo‐photocatalytic functionalities to achieve efficient antibacterial therapy and accelerated wound healing. Under simultaneous light and ultrasonic stimulation, the hydrogel exhibited robust piezo‐photocatalytic activity, achieving nearly complete inactivation of bacteria in vitro, significantly outperforming single‐mode treatments. Beyond its potent bactericidal effect, the PPBP hydrogel demonstrated excellent hemocompatibility and cytocompatibility, underscoring its biosafety for wound applications. In a *S. aureus*–infected wound model, synergistic piezoelectric stimulation and piezo‐photocatalytic therapy effectively suppressed bacterial proliferation, attenuated inflammatory responses, promoted epithelial regeneration, and enhanced collagen deposition, ultimately leading to rapid and nearly complete wound closure. The synergistic effect between piezoelectric and photocatalytic effects enables continuous transduction of mechanical and optical stimuli into catalytic activity, sustaining ROS generation and providing dynamic antibacterial action within the wound microenvironment. Collectively, this work establishes a versatile strategy for designing smart, self‐responsive, and biocompatible hydrogels that harness mechanical and optical energy to achieve synergistic therapeutic outcomes. The PPBP hydrogel not only represents a promising platform for infection control and tissue regeneration but also offers valuable insights for the development of next‐generation multifunctional wound dressings with noninvasive bioactivation.

## Author Contributions

Z. Lin, Y.N. Xie, and H. Dong supervised the project. B.Y. Dai. designed the research and wrote the manuscript. W.X. Qie prepared the materials, characterized carrier separation and migration behavior, and studied ROS generation property. X.Y. Li, J.B. Zheng and A. He conducted bacteria disinfection and wound healing experiments. J.J. Yan, H. Zhang, Z.H. Pan, H. Yin, C.Y. Zhou, R. Kong and H. Wang performed SEM, FT‐IR, and TEM characterizations. B.H. Dai polished the writing. The manuscript was written through contributions of all authors. All authors have given approval to the final version of the manuscript.

## Ethics Statement

All animal procedures and postoperative care were conducted in accordance with the guidelines of the Institutional Animal Care and Use Committee and were approved by the Animal Ethical and Welfare Committee of Nanjing University (Approval No. IACUC‐D2303104).

## Conflicts of Interest

The authors declare no conflicts of interest.

## Supporting information




**Supporting File**: advs77132‐sup‐0001‐SuppMat.docx.

## Data Availability

The data that supports the findings of this study are available in the supplementary material of this article.
