# Supplementary material for: Dual‐Responsive Hydrogels Integrate Bioelectrical Stimulation and Piezo‐Photocatalysis for Regenerative Treatment of Infected Wounds
Source: Adv Sci (Weinh). 2026 Aug 17;13(62):e77132. doi: 10.1002/advs.77132 (PMC13479854; doi:10.1002/advs.77132)
Supplement: Supplementary file 1 [file ADVS-13-e77132-s001.docx]

**Supporting information**

**Dual-Responsive Hydrogels Integrate Bioelectrical Stimulation and Piezo-Photocatalysis for Regenerative Treatment of Infected Wounds**

Baoying Dai^1,2^, Wenxuan Qie^1^, Xiaoye Li^3^, Jingben Zheng^3^, Ao He^3^, Heng Dong^3^*, Jiangjiang Yan^1^, Hao Zhang^1^, Ziheng Pan^1^, Hang Yin^1^, Chuyi Zhou^1^, Rui Kong^1^, Hao Wang^1^, Binghan Dai^4^, Yannan Xie^1^*, and Zhiqun Lin^2^*

^1^State Key Laboratory of Flexible Electronics (LoFE) and Institute of Advanced Materials (IAM), Jiangsu Provincial Industrial Technology Engineering Center for Flexible Mechatronics and Energy Systems, Jiangsu Key Laboratory of Smart Biomaterials and Theranostic Technology, Nanjing University of Posts and Telecommunications, Nanjing 210023, China.

^2^Department of Chemical and Biomolecular Engineering, National University of Singapore, Singapore 118425, Singapore

^3^Nanjing Stomatological Hospital, Affiliated Hospital of Medical School, Research Institute of Stomatology, Nanjing University, Nanjing 210008, China

^4^Affiliation: School of Foreign Studies, China University of Geosciences, Beijing, 100083, China

*Co-responding authors

E-mail: [dongheng90@smail.nju.edu.cn](mailto:dongheng90@smail.nju.edu.cn) (H. Dong); [iamynxie@njupt.edu.cn](mailto:iamynxie@njupt.edu.cn) (Y. Xie); [z.lin@nus.edu.sg](mailto:z.lin@nus.edu.sg) (Z. Lin)

# Experimental Section

**Materials**

Poly(vinylidene fluoride-co trifluoroethylene) copolymer (P(VDF-TrFE)) with 55% molar VDF content was purchased from Piezotech@Arkema. Dimethyl sulfoxide (DMSO, C_2_H_6_OS, 99.5%, Sinopharm Chemical Reagent Co. Ltd.) was used as solvent of P(VDF-TrFE). Bismuth nitrate pentahydrate (Bi(NO_3_)_3_‧5H_2_O, 99.99%), polyvinyl alcohol (PVA, Mw ~ 145000), sodium hydroxide (NaOH, 97%), acetic acid (C_2_H_4_O_2_, 99.5%), potassium chloride (KCl, 99.99%) and potassium bromide (KBr, 99%) from Shanghai Aladdin Biochemical Technology Co. Ltd. were used to synthesize BiOClBr. Dopamine hydrochloride (DA-HCl, 98%, Shanghai Aladdin Biochemical Technology Co. Ltd.) and trihydroxymethylaminomethane hydrochloride (Tris-HCl, Sangon Biotech Co. Ltd.) were employed to synthesize polydopamine (PDA). Isopropanol (IPA, C_3_H_8_O, 99.7%, Sinopharm Chemical Reagent Co. Ltd.), tryptophan (TRP, C_11_H_12_N_2_O_2_, 98%, Sigma-Aldrich), and benzoquinone (BQ, C_6_H_4_O_2_, 99.5%, Sigma-Aldrich) were obtained and specifically applied as scavengers for ·OH, ^1^O_2_, and ·O^2⁻^, respectively. 3,3′,5,5′-tetramethylbenzidine (TMB, C_16_H_20_N_2_, 99%, Sigma-Aldrich) and methyl orange (MO, C_14_H_14_N_3_NaO_3_S, 98%, Tianjin Chemical Reagent Research Institute Co. Ltd.) were employed as chromogenic probes for the detection of reactive oxygen species (ROS). All chemicals were used without additional purification. Deionized water and ethanol (C_2_H_5_OH, 99.7%, Wuxi City Yasheng Chemical Co. Ltd.) were used throughout the experimental procedures.

**Synthesis of BiOClBr**

BiOClBr was synthesized through a two-step process. First, Bi_2_O_3_ was prepared via a wet-chemical route. Specifically, 4.8 g of Bi(NO_3_)_3_‧5H_2_O was dissolved in 100 mL of deionized water and magnetically stirred at 500 rpm for 30 min at room temperature. Subsequently, NaOH solution was added dropwise until the pH reached 8.0. The resulting suspension was subjected to ultrasonic treatment for 60 min, followed by continuous magnetic stirring overnight. The obtained precipitate was collected by centrifugation, washed three times with ethanol and deionized water, and then dried at 60 °C for 24 h. Finally, the dried powder was calcined at 400 °C for 2 h to improve the crystallinity of Bi_2_O_3_.

In the second step, 1 mmol of the as-prepared Bi_2_O_3_ powder was dispersed in 10 mL of acetic acid solution and stirred for 20 min until the color of the suspension changed from yellow to white. Then, 30 mL of deionized water was added to obtain a transparent solution, followed by the addition of 1 mmol KCl and 1 mmol KBr. The mixture was stirred for an additional 30 min to complete the reaction. The resulting white precipitate was collected by filtration, thoroughly washed five times with ethanol and deionized water, and dried at 60 °C for 10 h to yield the final BiOClBr product.

**Preparation of BiOClBr@PDA**

1.0 g of the prepared BiOClBr powder was dispersed in 20 mL of Tris-HCl buffer solution and subjected to continuous ultrasonication at a frequency of 40 kHz for 30 min. Subsequently, 60 mg of DA-HCl particles was added, and the mixture was further ultrasonicated. The reaction was then allowed to proceed under stirring conditions at room temperature for 24 h, during which dopamine was polymerized and deposited on the surface of BiOClBr. After that, the solution was filtered and washed for three times, and the obtained precipitation was dried in an oven at 60 °C for 10 h to yield BiOClBr@PDA particles.

**Fabrication of PVA/P(VDF-TrFE)/BiOClBr@PDA (PPBP) Hydrogel**

1.75 g of PVA and 0.75 g of P(VDF-TrFE) was dissolved in 22.5 g of a mixed solvent consisting of DMSO and deionized water at a weight ratio of 9:1 at 90 °C with strong magnetic stirring to form homogeneous solution. After that, a certain amount (0, 0.05, 0.10, and 0.15 g) of the prepared BiOClBr@PDA powders were added into the solution. The mixture was subjected to ultrasonication for 20 min to promote uniform dispersion, followed by magnetic stirring to obtain a homogeneous suspension. Subsequently, the resulting mixture was cooled down to room temperature, poured into a mold (2.0×0.5 cm^2^), and frozen at -20 °C for 16 h, then thawed at room temperature for 8 h. This freeze–thaw process was repeated for two cycles. Finally, the obtained hydrogel was immersed in 1000 mL of distilled water for 2 days to remove residual DMSO, yielding PVA/P(VDF-TrFE) and PVA/P(VDF-TrFE)/BiOClBr@PDA (PPBP) composite hydrogel. For comparison, PVA hydrogel and P(VDF-TrFE) film were also prepared using the same method as that employed for fabricating the PPBP hydrogel. The only difference was that P(VDF-TrFE) and BiOClBr@PDA were not added for the PVA hydrogel preparation, and PVA and BiOClBr@PDA were not added for the P(VDF-TrFE) film preparation, respectively.

**Structure and Morphology Characterization**

Powder X-ray diffraction (XRD) measurements were carried out using an X-ray diffractometer (D8 Advance A25, Bruker) with Cu Kα radiation (λ = 0.154059 nm) at a scanning rate of 5 °/min to analyze the crystal structure of the as-prepared samples. X-ray photoelectron spectroscopy (XPS) was performed on an EX-250 spectrometer to examine the chemical states. Fourier transform infrared (FT-IR) spectra were recorded using a PerkinElmer Frontier spectrometer (PE-Spectrum Two) via the KBr pellet method to identify functional groups. Scanning electron microscopy (SEM, S4800, Hitachi), transmission electron microscopy (TEM, H7700, Hitachi) and high-resolution transmission electron microscopy (HRTEM, FEI Talos 200x, FEI) were used to observe the morphology of the samples. A UV-Vis-NIR spectrophotometer (Shimadzu 3101) was used to obtain diffuse reflectance spectra of the prepared samples, while a Lambda 35 UV-Vis spectrometer (UV 3600 Plus, PerkinElmer) was employed to observe the light absorption characteristics of MO and TMB solutions. The piezoelectric output performance of PVA, PVA/P(VDF-TrFE) and PPBP hydrogels under periodic deformation was evaluated using open-circuit potential time analysis. In addition, electron spin resonance (ESR) spectroscopy (EMXplus-6/1, Bruker) was employed to detect ROS generated by the composite hydrogels.

**Mechanical Property Test**

For the mechanical property test, the PPBP hydrogel was fabricated into a dumbbell-shaped specimen by directly casting the hydrogel precursor solution into a dumbbell-shaped mold, followed by freeze–thaw cycling. Tensile tests were performed using an electronic universal testing machine (I5-50N, Jinan Fangyuan Testing Instrument Co., Ltd.) at a stretching speed of 50 mm min⁻¹. The tensile stress and strain were calculated according to the following equations:

$$Stress（MPa）=\frac{F}{（W\times H）}$$

$$Strain=\frac{\Delta l}{l_{0}}$$

where $F$is the applied force, $W$and $H$represent the width and thickness of the specimen, respectively, $\Delta l$is the elongation of the sample during stretching, and $l_{0}$is the initial gauge length of the specimen.

**Rheological Characterization**

The rheological properties of the prepared PPBP hydrogel were characterized using a rotational rheometer (NETZSCH Kinexus). A parallel-plate geometry with a diameter of 40 mm was employed, and the gap between the plates was maintained at 1 mm. Distilled water was added around the sample to minimize solvent evaporation during the measurements. Oscillatory strain sweep tests were performed at 25 °C to evaluate the storage modulus (G′) and loss modulus (G″), with the shear strain ranging from 0.01% to 100%.

**Photothermal Behavior Characterization**

The photothermal behavior of the PPBP hydrogel was evaluated by monitoring the temperature variation under periodic light irradiation using an infrared thermal imaging camera (FOTRIC 226s, Shanghai Thermal Imaging Technology Co., Ltd.). The hydrogel samples were irradiated with a 200 W xenon lamp (CEL-HXF300-T3, Beijing China Education Au-Light Co., Ltd.) positioned at a distance of 26 cm. Periodic irradiation was applied with alternating light-on and light-off cycles of 4 min and 1 min, respectively, while the corresponding temperature changes were continuously recorded.

**Chemical Stability and Degradation Characterization**

To evaluate the chemical stability and degradation behavior of the prepared PPBP hydrogel under physiological conditions, the hydrogel samples were immersed in 10 mL of PBS solution (pH 7.4) and incubated for 15 days. The PBS solution was refreshed daily. At predetermined time intervals, the hydrogel samples were collected, freeze-dried, and weighed to determine the weight loss. The degradation ratio ($W_{d}$) was calculated according to the following equation:

$$W_{d}=\frac{W_{i}-W_{j}}{W_{i}}$$

where $W_{i}$represents the initial dry weight of the hydrogel, and $W_{j}$ is the dry weight of the hydrogel after incubation for a specific period.

In addition, XRD and FT-IR analyses were performed to investigate the structural stability of the PPBP hydrogel before and after immersion in PBS solution for 15 days.

**Photoelectrocatalytic Characterization**

The behavior of photoexcited charge carriers under various conditions was investigated using an electrochemical workstation (CHI 760E, Shanghai Chenhua Instrument Co., Ltd.) equipped with a three-electrode electrochemical system. Specifically, a platinum sheet, an Ag/AgCl electrode, and an indium tin oxide (ITO) glass electrode coated with the hydrogel sample were employed as the counter, reference, and working electrodes, respectively. A 200 W xenon lamp (CEL-HXF300-T3, Beijing China Education Au-Light Co., Ltd.) served as the light source and was positioned 5 cm below the electrolyte, which consisted of 40 mL of 0.5 M Na_2_SO_4_ solution (pH = 6.8). To monitor the carrier generation behavior of the PPBP samples under mechanical stress, an ultrasonic processor (180 W) was introduced into the measurement system in the absence of illumination. The piezo-photocurrent response and electrochemical impedance spectra of the PPBP samples were recorded under simultaneous irradiation with the xenon lamp and ultrasonic excitation. In addition, bias voltages of 0.5 V and 0.05 V were applied specifically for current output and electrochemical impedance measurements, respectively.

**ROS Capture Experiment and Catalytic Degradation Efficiency Characterization**

To detect the specific reactive species generated by the constructed PPBP hydrogel during photocatalysis, piezocatalysis, and their synergistic treatment process, TMB (0.1 M) and MO (2 mM) were selected as indicators to confirm the formation of ·OH and ^1^O_2_, and ·O_2_^-^, respectively. In addition, IPA (1.2 M), TRP (0.1 M), and BQ (1 M) dissolved in DMSO were employed as scavengers specifically targeting ·OH, ^1^O_2_, and ·O_2_^-^, respectively. Prior to illumination and ultrasonic irradiation, a piece of PPBP hydrogel (0.5 × 2 cm²) was immersed in 3 mL of the indicator solution, with or without the corresponding scavenger, and maintained in darkness to establish adsorption–desorption equilibrium. Specifically, to investigate the formation of ·OH and ·O_2_^-^, the PPBP hydrogel was immersed in TMB solution (3 mL) with or without 25 μL of IPA or 300 μL of TRP, followed by light and ultrasound co-irradiation for 1 h. The light absorption spectra of TMB solutions treated under the above-mentioned conditions were characterized to confirm the formation of ·OH and ·O_2_^-^. Furthermore, the degradation performance of MO (3 mL) catalyzed by the PPBP hydrogel was exclusively evaluated under light irradiation, ultrasound, and combined light and ultrasound conditions for 1 h, both in the presence and absence of 15 μL of BQ, to confirm ·O_2_^-^ generation. To quantitatively assess the ·O_2_^-^ yield on the PPBP hydrogel under these conditions, the UV-vis absorption spectra of MO solutions in the presence or absence of BQ were recorded at regular time intervals.

**Bacterial Inactivation Evaluation**

The photocatalytic disinfection experiments were performed using a xenon lamp (full-spectrum emission, light intensity 0.2 W cm^-2^) as the irradiation source, positioned 26 cm away from the reaction surface. The ultrasound irradiation groups were stimulated with ultrasound (1 MHz, 50% amplitude) at 1 W cm^-2^. The antibacterial performance against *Staphylococcus aureus* (*S. aureus*) was evaluated under five different conditions: (1) PBS solution (control), (2) PPBP hydrogel alone, (3) PPBP hydrogel under light irradiation, (4) PPBP hydrogel under ultrasonic stimulation, and (5) PPBP hydrogel under combined light and ultrasound irradiation. Light and ultrasound were alternately turned on for 4 min and off for 1 min over a total period of 30 min. After treatment, the surviving *S. aureus*, Methicillin-resistant *Staphylococcus aureus* (MRSA) and *Escherichia coli* (*E. coli*) in each group were quantified by the plate-counting method.

Additionally, live/dead bacterial staining of *S. aureus* was performed using a Calcein-AM/PI cytotoxicity assay kit, and fluorescence images were captured with a confocal laser scanning microscope (CLSM, Olympus FV1000MPE) to visualize bacterial viability at the microscopic level. The *S. aureus* samples from different treatment groups were fixed with 2.5% glutaraldehyde and then dehydrated by an ethanol gradient before being photographed by SEM.

***In vitro* Biofilm Clearance Assay**

Bacterial biofilms were established by incubating bacterial suspensions in 6-well plates at 37 °C for 24 h, followed by gentle washing with PBS to remove planktonic bacteria. To avoid direct mechanical disruption of the biofilm, a non-contact Transwell-like setup was used, in which PPBP hydrogel was placed on a sterile mesh above the mature biofilm. The samples were divided into six groups: PBS, PBS + light + ultrasound, PPBP, PPBP + light, PPBP + ultrasound, and PPBP + light + ultrasound. After the corresponding treatments, the remaining biofilms were washed, fixed, and stained with crystal violet. Representative images were captured under an optical microscope. For quantitative analysis, the bound crystal violet was dissolved, and the absorbance was measured at 590 nm to determine the relative biofilm biomass.

***In Vivo* Infected Wound Treatment**

All animal procedures and postoperative care were conducted in accordance with the guidelines of the Institutional Animal Care and Use Committee and were approved by the Animal Ethical and Welfare Committee of Nanjing University (approval no. IACUC-D2303104). Male BALB/c mice (6 weeks old) were anesthetized prior to the creation of full-thickness circular wounds on the dorsal skin. The wounds were then infected with a suspension of S. aureus to establish an infected wound model. Subsequently, PBS or PPBP composite hydrogels were applied to the wound sites and divided into five treatment groups: (1) PBS, (2) PPBP hydrogel without stimulation, (3) PPBP hydrogel under light irradiation, (4) PPBP hydrogel under ultrasonic stimulation, and (5) PPBP hydrogel under combined light and ultrasound treatment. For light stimulation, a xenon lamp (current 9 A) was positioned 37 cm above the mice and irradiated for 4 min with 1 min intervals of rest, while simultaneous ultrasonic stimulation (1 MHz, 50% amplitude, 1 W cm^-2^) was applied to the dorsal region to mimic natural skin deformation and induce piezoelectric excitation. Wound images were recorded on days 0, 1, 3, 5, 7, and 9, and the wound area was quantified using ImageJ software. On day 9, wound tissues were harvested and homogenized in 1 mL of PBS, and 100 μL of each suspension was spread onto agar plates and incubated at 37 °C for 18 h. The formation and number of *S. aureus* colonies were then observed and quantitatively analyzed to evaluate antibacterial efficacy and wound healing performance.

***In situ* ROS Staining of Wound Tissues**

To evaluate local ROS generation *in vivo*, wound tissues were collected from mice at 12 h after treatment, fixed, embedded, and sectioned. The tissue sections were stained with a ROS-sensitive fluorescent probe to detect ROS production, and nuclei were counterstained with DAPI. Fluorescence images were captured using a fluorescence microscope, and the mean fluorescence intensity of ROS was quantified to compare ROS levels among different treatment groups.

**qRT-PCR Analysis of Wound Tissues**

Wound skin tissues were collected from BALB/c mice on Day 9 after treatment. The wound tissues, including the central wound area and approximately 1~2 mm of surrounding skin margin, were excised, immediately frozen in liquid nitrogen, and stored at −80 °C until RNA extraction. Primer sequences for qRT-PCR were listed in **Table S1**. Total RNA was isolated using TRIzol reagent according to the manufacturer’s instructions. The concentration and purity of RNA were determined by measuring the absorbance at 260 and 280 nm. Subsequently, equal amounts of total RNA were reverse-transcribed into cDNA using a reverse transcription kit. Quantitative real-time PCR was performed using SYBR Green qPCR Master Mix on a real-time PCR system. The relative mRNA expression levels of *Tgfb1*, *Arg1*, *Vegfa*, and *Col1a1* were normalized to *Gapdh* and calculated using the 2^−ΔΔCt^ method.

**Histological Examination**

On day 9, the BALB/c mice were euthanized, and the wound tissues were collected for histological analysis. The samples were fixed in 4% paraformaldehyde, embedded in paraffin, and sectioned. Hematoxylin and eosin (H&E) staining and Masson staining were performed to evaluate inflammatory infiltration, epithelial regeneration, and collagen deposition at the wound sites.

To evaluate the effect and safety of different ultrasonic stimulation intensities, PPBP hydrogels were applied under combined light irradiation and ultrasonic stimulation at different intensities, including 0, 0.5, 1, and 2 W/cm^2^. For angiogenesis evaluation, regenerated wound tissues were collected on Day 9 after treatment, fixed, embedded, and sectioned. The sections were stained with anti-CD31 antibody to label vascular endothelial cells, and nuclei were counterstained with DAPI. Fluorescence images were captured using a fluorescence microscope, and the mean fluorescence intensity of CD31 was quantified to assess angiogenesis under different stimulation conditions.

For skin safety evaluation, normal skin tissues were exclusively exposed to ultrasonic stimulation at 0.5, 1, and 2 W/cm^2^, with untreated skin used as the control. After treatment, the skin tissues were collected, fixed, embedded, sectioned, and stained with H&E. Histological images were observed to evaluate potential epidermal damage, dermal necrosis, inflammatory infiltration, and subcutaneous tissue injury caused by different ultrasonic intensities.

**Routine Blood Analysis and Bi Biodistribution Assay**

To evaluate the long-term biosafety of PPBP hydrogel, blood samples were collected from mice after different treatments for routine hematological analysis. The levels of white blood cells (WBC), red blood cells (RBC), monocytes (MON), mean corpuscular volume (MCV), platelets (PLT), and lymphocytes (LYM) were measured using an automated hematology analyzer on Day 14 and compared with normal physiological reference ranges.

For Bi biodistribution analysis, major organs, including the heart, liver, spleen, lung, and kidney, were collected on Day 14 and Day 28 after treatment. The organ samples were weighed, digested with acid solution, and the Bi concentration was quantified by inductively coupled plasma mass spectrometry (ICP-MS). The Bi content in each organ was expressed as mg/kg tissue.

**Statistical Analysis**

The data were expressed as the mean ± SD. Intergroup and intragroup comparison analyses in each experiment were calculated through one- or two-way ANOVA with a Tukey’s post-hoc test by using GraphPad Prism. *p*-values < 0.05 were considered statistically significant.

**2. Supplementary Figures and Table**

**Figure** **S1.** FT-IR spectra of BiOClBr and BiOClBr@PDA powders, and PVA hydrogel, P(VDF-TrFE) film and PVA/P(VDF-TrFE) hydrogel.

**Figure** **S2.** The storage modulus (G′) and loss modulus (G′′) curves of the PPBP hydrogel.

**Figure S3.** Stress–strain curve of the PPBP hydrogel obtained from the uniaxial tensile test.

**Figure S4.** The weight losses of the PPBP hydrogel during immersion in PBS solution for 15 days.

**Figure S5.** (a) XRD patterns and (b) FT-IR spectra of the PPBP hydrogel detected before and after immersed in PBS solution for 15 days.

**Figure S6.** Digital photographs showing the detachment process of the PPBP hydrogel from the skin under water rinsing after immersion in PBS solution for 15 days.

**Figure S7.** Piezoelectric potential output of the PPBP hydrogel under a uniform compression stress induced by a 200 g weight from a height of 5 cm.

**Figure S8.** Light absorption curves of MO solution (3mL, 1mg/mL) observed before and after treated with the PPBP hydrogel under (a) light and (b) ultrasonic stimulation in the presence (MO+BQ-1h) or absence (MO-1h) of BQ.

**Figure S9.** XRD patterns of the PPBP hydrogels incorporating with different contents of BiOClBr@PDA.

**Figure S10.** SEM images of the PPBP hydrogels containing (a) 0.05 g and (b) 0.15 g of BiOClBr@PDA.

**Figure S11.** Degradation efficiency of MO solution (3mL, 1mg/mL) catalyzed by PPBP hydrogels with different BiOClBr@PDA contents under the combined light and ultrasonic stimulation.

**Figure S12.** The temperature variation curve of the PPBP hydrogel recorded under the periodic light on and off conditions.

**Figure S13. Antibacterial efficiency for MRSA and *E. coli*.** (a) Photographs of MRSA and *E. coli* colonies in different groups. Quantitative analysis of viable (b) MRSA and (c) *E. coli* colonies calculated from (a). Groups: PBS (Group 1), PPBP hydrogel without stimulation (Group 2), PPBP hydrogel under light irradiation (Group 3), PPBP hydrogel under ultrasonic stimulation (Group 4), and PPBP hydrogel under combined light and ultrasonic irradiation (Group 5). Data are presented as mean ± SD. Statistical significance was analyzed by one-way ANOVA. *****p* < 0.0001.

**Figure S14.** (a) Schematic diagram and photographs for *in vitro* bacterial biofilm model**.** (b) Microscope images of biofilms stained by crystal violet in different groups. (c) Relative biofilm biomass measured by crystal violet-stained biofilms. Data are presented as mean ± SD. Statistical significance was analyzed by one-way ANOVA. *****p* < 0.0001.

**Figure S15.** ***In situ* detection of ROS generation in wound tissues.** (a) Representative fluorescence images of wound tissue sections collected 12 h after treatment and stained with DAPI and a ROS-sensitive probe. Blue fluorescence indicates DAPI-stained nuclei, and red fluorescence indicates ROS accumulation within the wound tissue. (b) Quantitative analysis of ROS-associated fluorescence intensity in different treatment groups. Data are presented as mean ± SD. ****p* < 0.001, *****p* < 0.0001.

**Figure S16.** **Quantitative analysis of collagen deposition in regenerated wound tissues.** Collagen deposition in wound tissues was quantified based on histological staining images. Data are presented as mean ± SD. *****p* < 0.0001.

**Figure S17.** **Expression of tissue repair-related genes in wound tissues on Day 9.** Wound tissues were collected on Day 9 after treatment, and the relative mRNA expression levels of (a) *Tgfb1*, (b) *Arg-1*, (c) *Vegfa*, and (d) *Col1a1* were analyzed by qRT-PCR. Data are presented as mean ± SD. ****p* < 0.001, *****p* < 0.0001

**Figure S18. Evaluation of angiogenesis in regenerated wound tissues under different ultrasonic stimulation intensities**. (a) Representative immunofluorescence images of wound tissue sections stained with DAPI and CD31 after treatment under different ultrasonic intensities, including 0, 0.5, 1, and 2 W/cm². Blue fluorescence indicates DAPI-stained nuclei, and red fluorescence indicates CD31-positive vascular endothelial cells. (b) Quantitative analysis of the mean fluorescence intensity of CD31 in different groups. Data are presented as mean ± SD. Statistical significance was analyzed by one-way ANOVA. ****p* < 0.001, *****p* < 0.0001.

**Figure S19.** H&E-stained sections of major organs. The heart, liver, spleen, lung, and kidney from mice treated with either PBS (Group 1) or PPBP hydrogel under the combined light and ultrasonic excitation (Group 5).

**Figure S20. Routine blood analysis after different treatments**. Hematological parameters of mice in different treatment groups were evaluated, including (a) WBC, (b) RBC, (c) MON, (d) MCV, (e) PLT, and (f) LYM. The gray shaded regions indicate the corresponding normal physiological reference ranges. Data are presented as mean ± SD.

**Figure S21.** **Biodistribution of Bi in major organs after the PPBP hydrogel treatment.** Bi concentrations in the heart, liver, spleen, lung, and kidney were quantified on (a) Day 14 and (b) Day 28 after treatment. Data are presented as mean ± SD.

**Figure S22. Histological evaluation of normal skin tissues after ultrasonic stimulation with different intensities.** Representative H&E-stained images of normal skin tissues after treatment with different ultrasonic intensities, including control, 0.5, 1, and 2 W/cm^2^. No obvious epidermal damage, dermal necrosis, inflammatory infiltration, or subcutaneous tissue injury was observed in any ultrasonic stimulation group.

**Table S1. Primer sequences used for RT-qPCR**

| **Genes** | **Forward primer (5**′**–3**′**）** | **Reverse primer (5**′**–3**′**）** |
| --- | --- | --- |
| *Tgfb1* | CTCCCGTGGCTTCTAGTGC | GCCTTAGTTTGGACAGGATCTG |
| *Arg1* | CTCCAAGCCAAAGTCCTTAGAG | AGGAGCTGTCATTAGGGACATC |
| *Vegfa* | GCACATAGAGAGAATGAGCTTCC | CTCCGCTCTGAACAAGGCT |
| *Col1a1* | GCTCCTCTTAGGGGCCACT | CCACGTCTCACCATTGGGG |
| *Gapdh* | AGGTCGGTGTGAACGGATTTG | TGTAGACCATGTAGTTGAGGTCA |
